# Antithyroid Drugs in the Management of Graves' Disease: A Friend and Foe

**DOI:** 10.7759/cureus.36028

**Published:** 2023-03-11

**Authors:** Muhammad I Butt, Muhammad Riazuddin, Faisal Joueidi, Najeeb Waheed

**Affiliations:** 1 College of Medicine, Alfaisal University, Riyadh, SAU; 2 Medicine, King Faisal Specialist Hospital and Research Centre, Riyadh, SAU; 3 Endocrinology and Diabetes, Imperial College London Diabetes Centre, Al Ain, ARE

**Keywords:** propylthiouracil, methimazole, trab, fetal thyrotoxicosis, thyroidectomy, antithyroid agents, hyperthyroidism, graves disease

## Abstract

Graves' disease is an autoimmune condition in which the patient develops autoantibodies that stimulate the thyroid gland, leading to thyrotoxicosis. We report the case of a 29-year-old female who presented one month postpartum with typical symptoms and signs of thyrotoxicosis. Biochemical and radiological investigations confirmed thyrotoxicosis due to Graves' disease. She received methimazole (MMI) treatment, leading to an allergic reaction in the form of a generalized rash on the body precluding its use. We later started the treatment with propylthiouracil, which she initially tolerated well. During her treatment, she became pregnant and delivered a baby girl by cesarean section at 37 weeks of gestation. The baby developed neonatal thyrotoxicosis due to the transplacental transmission of maternal thyrotropin receptor antibodies. Thyrotoxicosis was short-lived, without consequences, and treated with antithyroid drugs. Three months after delivery, thyroid hormone levels rose considerably, requiring higher doses of propylthiouracil, which resulted in severe hepatic dysfunction, and therefore we stopped the therapy. We admitted her to the hospital for rapid correction of thyroid hormones using steroids, supersaturated potassium iodide, and cholestyramine before she underwent a total thyroidectomy. Our case highlights the challenges the patients and clinicians can face while managing Graves' disease. We discuss the role of a multidisciplinary team approach to care and the options available for treatment in such difficult situations.

## Introduction

Thyroid gland disorders are common and affect a considerable proportion of the population, with females, affected more often than men [1-3]. Graves' disease (GD) is the commonest cause of thyrotoxicosis in young- and middle-aged women. GD is an autoimmune condition characterized by increased production of thyrotropin receptor antibodies (TRAbs) that stimulate the thyroid gland, leading to thyroid hormone excess. There are different options for the treatment of GD. These modalities include antithyroid drugs (ATDs), radioactive iodine (RAI) ablation of the thyroid gland, and total thyroidectomy (TT). The treatment depends on patient preference and physician recommendations, and in most cases, patients elect to use ATD as their first choice, with a treatment course lasting 12-18 months. However, a proportion of patients take the 40-50% risk of relapse upon stopping the ATD as a deterrent for pursuing this option. Elderly patients and those with pre-existing cardiac comorbidities prefer RAI. Patients with large goiters and pressure symptoms or those who have experienced relapse following cessation of the course of the antithyroid drugs benefit from TT [4].

## Case presentation

We report a 29-year-old female with GD who encountered various challenges during treatment. She presented to her local hospital with a four-month history of anxiety, tremors, sweating, palpitations, 4-kg weight loss, and painless anterior neck swelling. These symptoms started when she was one month postpartum. She was otherwise fit and well, with no significant past medical history. There was no family history of thyroid disorders. The clinical examination revealed fine tremors in both hands, a pulse of 118 beats per minute, and a blood pressure of 118/58 mmHg. Her body weight was 58 kg, with a body mass index of 22.8 kg/m². She had a sizeable diffuse goiter involving both lobes of the thyroid gland. Thyroid bruit was heard over the thyroid gland. She had Graves' ophthalmopathy, as evidenced by chemosis and proptosis of both eyes.

Blood tests confirmed thyrotoxicosis due to GD with strongly positive TRAb (Table 1).

**Table 1:** Baseline thyroid and liver function tests.

	Results	Reference range
Free T4 (FT4)	77.4 pmol/L	12-22 pmol/L
Thyroid-stimulating hormone (TSH)	<0.01 mlU/L	0.27-4.2 mlU/L
TSH receptor antibody (TRAb)	>36.0 IU/L	<1.8 IU/L
Total bilirubin	3.6 µmol/L	0-21 µmol/L
Alanine transaminase (ALT)	37.6 U/L	10-45.0 U/L
Aspartate aminotransferase (AST)	54 U/L	10-45.0 U/L
Alkaline phosphatase (ALP)	137 U/L	46.0-122.0 U/L

**Figure 1:**
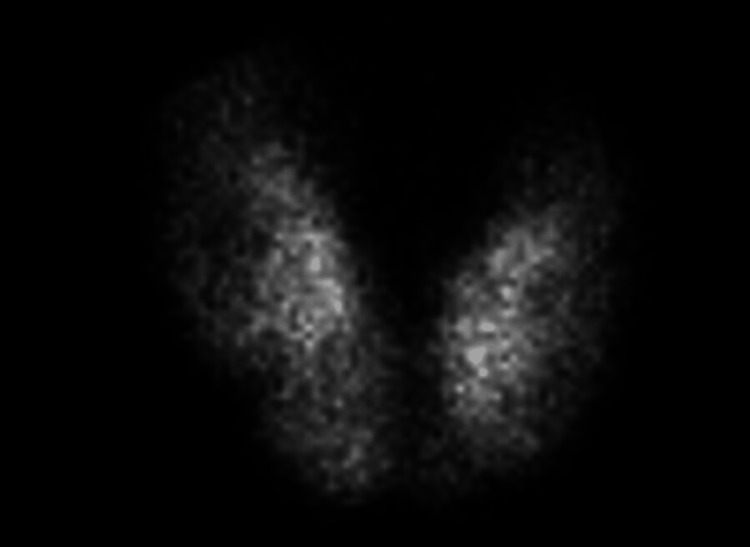
Iodine-123 (I-123) thyroid uptake scan.

A thyroid iodine-123 (I-123) uptake scan showed bilaterally increased radiotracer uptake, excluding toxic nodules and thyroiditis as the causes of thyrotoxicosis (Figure 1).

We offered her the choice of either RAI ablation or TT. She was inclined to try propylthiouracil (PTU) before considering permanent treatment options. She acknowledged the risk of cross-reactivity and similar adverse effects with its use. We started PTU 100 mg twice a day (BID) and checked her liver function tests at baseline (Table 1). These were slightly deranged, as expected in patients with thyrotoxicosis [5]. We titrated the dose as needed during treatment.

She missed the in-person and virtual appointments during the COVID-19 restrictions, which posed difficulties in providing timely care. During her subsequent visit, she reported being three months pregnant. We monitored her thyroid and hepatic functions and adjusted her dose as required. She did not return to the clinic until three months postpartum. She was taking PTU 100 mg BID. Thyroid-stimulating hormone (TSH) was <0.01 mU/L, and free thyroxine (FT4) was 33 pmol/L. 

She informed us that she had delivered a baby girl, pre-term at 37 weeks of gestation, by cesarean section at her local hospital. The baby had a birth weight of 2.91 kg, and medical staff noticed symptoms of thyrotoxicosis with palpitations and sweating two days after her birth. The pediatrician treated her with methimazole (MMI) 2.5 mg daily. We do not have her baseline thyroid function tests from the regional hospital.

**Figure 2:**
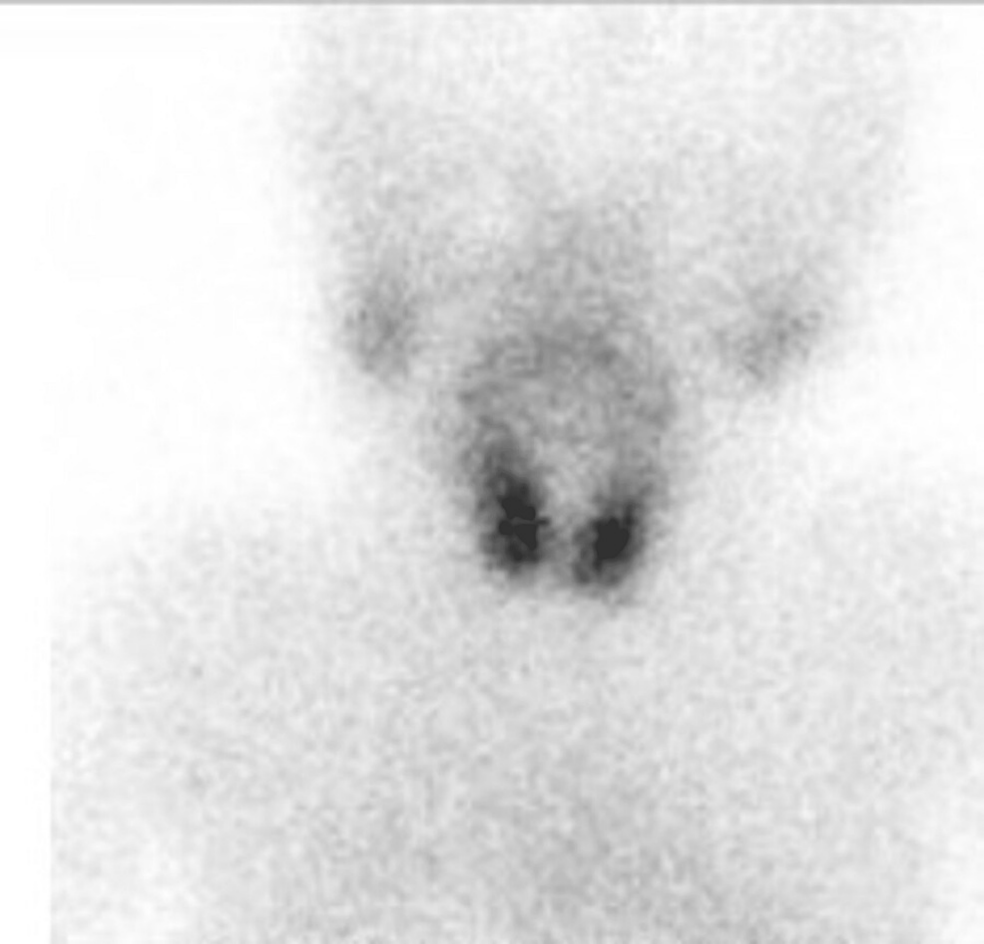
Iodine-123 (I-123) thyroid uptake scan of the baby.

We increased the dose of PTU of our patient to 100 mg three times a day (TID) and referred her baby to the pediatric endocrinologist at our institution. They arranged the thyroid uptake scan of the baby, which was entirely normal, excluding the possibility of congenital GD and confirming the transplacental transmission of maternal TRAb as the cause of neonatal thyrotoxicosis (Figure 2). The baby is well and is no longer on ATD and is euthyroid.

**Figure 3:**
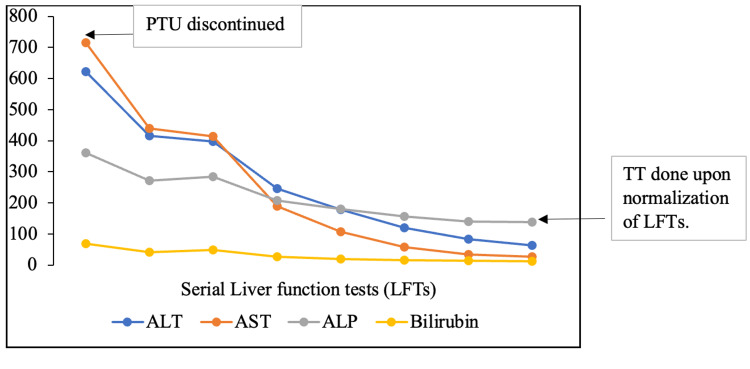
Improvement of LFTs after cessation of propylthiouracil (PTU). AST: aspartate transaminase (reference range: 10.0-45.0 U/L), ALT: alanine transaminase (reference range: 10.0-45.0 U/L), ALP: alkaline phosphatase (reference range: 46.0-122.0 U/L), bilirubin (reference range: 0.0-21.0 µmol/L), TT: total thyroidectomy, PTU: propylthiouracil, LFTs: liver function tests.

Our patient returned to the clinic after a month with complaints of itchiness, jaundice, and dark urine color for two weeks. She had evidence of severe hepatic dysfunction (Figure 3).

We stopped her PTU and admitted her for consideration of TT as she was biochemically euthyroid with TSH <0.01 mU/L and FT4 of 18 pmol/L. We arranged a multidisciplinary team meeting involving the hepatologist and thyroid surgeon. It was deemed a high surgical risk due to hepatic dysfunction. The nature of hepatic dysfunction was unclear and could have been due to GD, ATD, or previously undiagnosed primary liver disease. We deferred the surgery for one week and arranged a liver ultrasound, hepatic viral serology, and immunology studies (Table 2).

**Table 2:** Hepatic serology.

	Results	Reference range
Hepatitis A, immunoglobulin M (IgM) antibody	Non-reactive	-
Hepatitis A, total antibody	Reactive	-
Hepatitis Bs antigen	Non-reactive	-
Hepatitis Bs total antibody	Reactive	-
Hepatitis Bs total antibody	Reactive	-
Hepatitis Bc total antibody	Non-reactive	-
Hepatitis Bc immunoglobulin M (IgM) antibody	Non-reactive	-
Hepatitis C virus antibody	Non-reactive	-
Hepatitis E virus IgG	Non-reactive	-
Hepatitis E virus immunoglobulin M (IgM) antibody	Non-reactive	-
Hepatitis delta antibody	Non-reactive	-
Antinuclear antibody (ANA)	Negative	-
Anti-smooth muscle antibody (ASMA)	Negative	-
Anti-tissue transaminase immunoglobulin A (IgA)	7.0	0–20 U/ml
Anti-reticulin immunoglobulin A (IgA)	Negative	-
Anti-endomysium immunoglobulin A (IgA)	Negative	-
Anti-mitochondrial antibody (AMA)	Negative	-
Immunoglobulin G (IgG)	14.3 g/L	7.0–16.0 g/L
Immunoglobulin A (IgA)	3.13 g/L	0.70–4.00 g/L
Immunoglobulin M (IgM)	2.32 g/L	0.40–2.30 g/L

**Figure 4:**
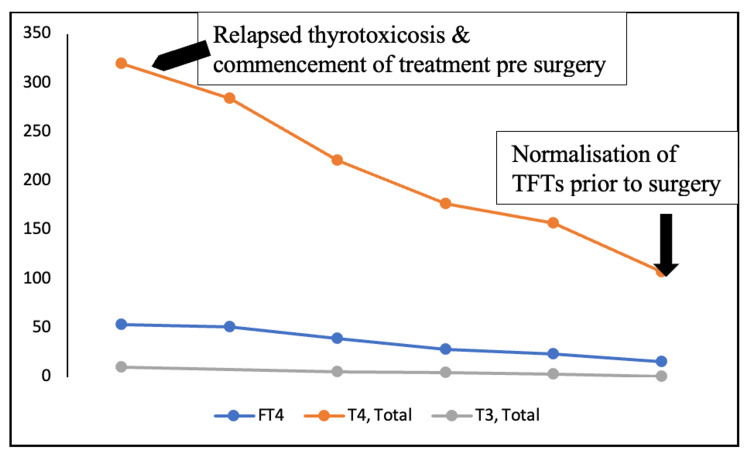
Serial thyroid function tests (TFTs) before surgery. FT4: free thyroxine (reference range: 12-22 pmol/L), T4, total: thyroxine (reference range: 66.0-181.0 nmol/L), and T3, total: triiodothyronine (reference range: 1.3-3.1 nmol/L).

We admitted her to our ward for surgery after the hepatologists completed their radiological and biochemical evaluations, which were all normal. However, now she posed a more significant challenge with a relapse of thyrotoxicosis (Figure 4).

We initiated MMI 20 mg BID once again under direct supervision. There was a risk of an allergic reaction, which she had experienced before. However, this treatment was necessary due to a relapse of thyrotoxicosis. In addition, we started propranolol 40 mg TID, supersaturated potassium iodide (SSKI) six drops BID, prednisolone 40 mg once a day (OD), and cholestyramine 4 g TID. She attained euthyroidism within four days without experiencing any side effects due to MMI (Figure 4) and improved liver function tests (Figure 3). We performed TT, which went well without any surgical complications. Histopathology confirmed diffuse hyperplasia of the thyroid gland consistent with GD. She takes levothyroxine 100 mcg OD, which has rendered her euthyroid.

## Discussion

Our patient presented multiple challenges during her treatment for GD. These included being unable to attend the clinic appointment for ongoing evaluations, monitoring thyroid functions and dose titration due to COVID-19 restrictions, experiencing the rare allergic reaction to MMI, the development and progression of hepatic dysfunction due to PTU, postponement of surgery due to hepatic dysfunction, relapse of thyrotoxicosis following cessation of PTU, and finally the development of neonatal thyrotoxicosis. 

Thyrotoxicosis is well-recognized to cause hepatic dysfunction by inducing apoptosis of the hepatocytes [5]. Similarly, hepatic dysfunction due to ATD is a recognized adverse effect. It is seen more often with PTU than MMI (6.3% vs. 1.4%), with an overall prevalence of 2.5% [6]. The underlying mechanism of hepatotoxicity is related to the direct toxic action of the drug and its metabolites on the hepatocytes and the immune-mediated response mediated by the cytokines, T cells, and interleukin 4 that leads to hepatic necrosis [7,8].

The immunity is physiologically downregulated during pregnancy to avoid rejecting the fetus and for the pregnancy to progress safely. In the postpartum period, changes in the immune system can lead to worsening autoimmune conditions [9]. In our patient, this manifested in the form of rising thyroid hormones and an increase in the dose requirement of ATD that eventually led to hepatotoxicity [10].

During her admission, we used multiple lines of treatment working on various pathways, which worked in synergy to render her euthyroid rapidly. We used steroids to reduce the secretion of thyroid hormones due to their direct effect on the underlying immune process and inhibition of peripheral FT4 to active free triiodothyronine (FT3) conversion [11]. Patel et al. [12] reported a similar case with Graves' disease, significant thyroid eye disease, and hepatic dysfunction. He was able to use steroids to manage all the consequences of GD, including Graves' ophthalmopathy, thyrotoxicosis, and hepatic dysfunction.

We added SSKI, which reduces the thyroid gland's vascularity, making surgery safe. Moreover, it impairs the synthesis and release of thyroid hormones. Additionally, it helps to use lower doses of ATD, which is particularly useful in cases such as our patient who experienced an allergic reaction to these agents [13].

Er et al. [14] treated their patient with Graves' disease with anti-thyroid drugs leading to agranulocytosis, limiting their use. They had to use cholestyramine as the sole therapy for treating Graves' disease, which led to euthyroidism within a few days. In contrast, we added cholestyramine to ATDs, which lowers thyroid hormone levels by binding to thyroid hormones and enhancing the excretion of the thyroid hormones in feces. In addition, these drugs work synergistically with ATD to achieve euthyroidism faster than ATD alone [15,16].

The baby suffered neonatal thyrotoxicosis induced by the transplacental transmission of maternal TRAb. The risk of neonatal thyrotoxicosis is higher in patients such as our patient with persistently higher TRAb in the third trimester of pregnancy and those patients who continue to require ATD toward the end of pregnancy and therefore require close monitoring [17,18]. We could not undertake this as the patient did not attend the clinic from the first trimester of pregnancy till three months postpartum.

## Conclusions

Our case emphasizes the importance of classic treatment protocols for Graves' disease. Our case serves as a reminder that patients with GD need close follow-up and monitoring of the thyroid function tests and for any adverse effects such as allergic reactions and hepatic dysfunction. It also highlights the importance of monitoring the babies of mothers with GD for signs and symptoms of thyroid disorder at birth to ensure prompt care.

## References

[1] Bjoro T, Holmen J, Krüger O (2000). Prevalence of thyroid disease, thyroid dysfunction and thyroid peroxidase antibodies in a large, unselected population. The Health Study of Nord-Trondelag (HUNT). Eur J Endocrinol.

[2] Reiners C, Wegscheider K, Schicha H, Theissen P, Vaupel R, Wrbitzky R, Schumm-Draeger PM (2004). Prevalence of thyroid disorders in the working population of Germany: ultrasonography screening in 96,278 unselected employees. Thyroid.

[3] Garmendia Madariaga A, Santos Palacios S, Guillén-Grima F, Galofré JC (2014). The incidence and prevalence of thyroid dysfunction in Europe: a meta-analysis. J Clin Endocrinol Metab.

[4] Kahaly GJ, Bartalena L, Hegedüs L, Leenhardt L, Poppe K, Pearce SH (2018). 2018 European Thyroid Association guideline for the management of Graves' hyperthyroidism. Eur Thyroid J.

[5] Kumar A, Sinha RA, Tiwari M (2007). Hyperthyroidism induces apoptosis in rat liver through activation of death receptor-mediated pathways. J Hepatol.

[6] Suzuki N, Noh JY, Hiruma M (2019). Analysis of antithyroid drug-induced severe liver injury in 18,558 newly diagnosed patients with Graves' disease in Japan. Thyroid.

[7] Kobayashi M, Higuchi S, Ide M, Nishikawa S, Fukami T, Nakajima M, Yokoi T (2012). Th2 cytokine-mediated methimazole-induced acute liver injury in mice. J Appl Toxicol.

[8] Heidari R, Niknahad H, Jamshidzadeh A, Eghbal MA, Abdoli N (2015). An overview on the proposed mechanisms of antithyroid drugs-induced liver injury. Adv Pharm Bull.

[9] Davies TF (1999). The thyroid immunology of the postpartum period. Thyroid.

[10] Wang MT, Lee WJ, Huang TY, Chu CL, Hsieh CH (2014). Antithyroid drug-related hepatotoxicity in hyperthyroidism patients: a population-based cohort study. Br J Clin Pharmacol.

[11] Brancatella A, Pierotti L, Viola N (2022). Steroid treatment in the management of destructive thyrotoxicosis induced by PD1 blockade. Eur Thyroid J.

[12] Patel AM, Stanback C, Vellanki P (2021). Clinical case report: dissociation of clinical course of coexisting autoimmune hepatitis and Graves disease. AACE Clin Case Rep.

[13] Tsai CH, Yang PS, Lee JJ, Liu TP, Kuo CY, Cheng SP (2019). Effects of preoperative iodine administration on thyroidectomy for hyperthyroidism: a systematic review and meta-analysis. Otolaryngol Head Neck Surg.

[14] Er C, Sule AA (2016). Cholestyramine as monotherapy for Graves' hyperthyroidism. Singapore Med J.

[15] Tsai WC, Pei D, Wang TF (2005). The effect of combination therapy with propylthiouracil and cholestyramine in the treatment of Graves' hyperthyroidism. Clin Endocrinol (Oxf).

[16] Kaykhaei MA, Shams M, Sadegholvad A, Dabbaghmanesh MH, Omrani GR (2008). Low doses of cholestyramine in the treatment of hyperthyroidism. Endocrine.

[17] Besançon A, Beltrand J, Le Gac I, Luton D, Polak M (2014). Management of neonates born to women with Graves' disease: a cohort study. Eur J Endocrinol.

[18] Luz IR, Martins JR, Jerónimo M, Caetano JS, Cardoso R, Dinis I, Mirante A (2020). Neonates born to mothers with Graves' disease: 15 year experience of a pediatric endocrinology department. Acta Med Port.

